# Patient navigators for people with chronic disease: protocol for a systematic review and meta-analysis

**DOI:** 10.1186/s13643-015-0019-1

**Published:** 2015-03-14

**Authors:** Elizabeth Kelly, Noah Ivers, Rami Zawi, Lianne Barnieh, Braden Manns, Diane L Lorenzetti, David Nicholas, Marcello Tonelli, Brenda Hemmelgarn, Richard Lewanczuk, Alun Edwards, Ted Braun, Kerry A McBrien

**Affiliations:** Department of Family Medicine, University of Calgary, G012, Health Sciences Centre, 3330 Hospital Drive NW, Calgary, AB T2N 4 N1 Canada; Department of Family and Community Medicine, Women’s College Hospital, University of Toronto, 77 Grenville Street 4th Floor, Toronto, Ontario M5S 1B3 Canada; Department of Medicine, University of Calgary, 1403 29th Street NW, Calgary, AB T2N 2 T9 Canada; Department of Community Health Sciences, University of Calgary, TRW Building, 3280 Hospital Drive NW, Calgary, AB T2N 4Z6 Canada; Faculty of Social Work, University of Calgary, 2800 University Way NW, Calgary, AB T2N 1 N4 Canada; Department of Medicine, University of Alberta, 362 HMRC, Edmonton, AB T6G 2S2 Canada; Alberta Health Services, 10030-107 Street NW, Edmonton, AB T5J 3E4 Canada; Department of Family Medicine, Alberta Health Services, Sheldon M. Chumir Health Centre, 8th Floor, 1213 – 4th Street SW, Calgary, AB T2R 0X7 Canada

**Keywords:** Patient navigator, Chronic disease, Systematic review

## Abstract

**Background:**

Individuals with chronic diseases may have difficulty optimizing their health and getting the care they need due to a combination of patient, provider, and health system level barriers. Patient navigator programs, in which trained personnel assess and assist patients in overcoming barriers to care, may improve care and outcomes for patients with chronic disease by providing an alternative approach to conventional information and support resources.

**Methods:**

This systematic review will evaluate the evidence for patient navigator programs, compared to usual care, in patients with chronic disease. We will include RCTs, cluster RCTs, and quasi-randomized RCTs that study the effects of patient navigator programs on clinical outcomes, patient experience, and markers of adherence to care. Studies will be identified by searching MEDLINE, Embase, the Cochrane Central Register of Controlled Trials (CENTRAL), CINAHL, PsycINFO, Social Work Abstracts, and the references of included studies. Two authors will screen titles and abstracts independently. Full texts will be reviewed for relevance and data extraction will be done independently by two authors. Studies will be included if they assess patients of any age with one or more chronic diseases. Outcomes will be categorized into groups characterized by their proximity to mechanism of action of the intervention: patient-level outcomes, intermediate outcomes, and process outcomes. Descriptive data about the elements of the patient navigator intervention will also be collected for potential subgroup analyses. Risk of bias will be assessed using the Effective Practice and Organisation of Care Group (EPOC) risk of bias tool. Data will be analyzed using random effects meta-analysis (relative risk for dichotomous data and mean difference for continuous data), if appropriate.

**Discussion:**

A comprehensive review of patient navigator programs, including a summary of the elements of programs that are associated with a successful intervention, does not yet exist. This systematic review will synthesize the evidence of the effect of patient navigator interventions on clinical and patient-oriented outcomes in populations across a comprehensive set of chronic diseases.

**Systematic Review Registration:**

PROSPERO CRD42013005857.

## Background

Chronic diseases are a significant burden to patients and the health care system. They include both physical and mental illnesses and affect at least one third of all Canadians; of Canadian adults with a chronic disease, over a third have two or more conditions [1]. People with chronic conditions have increased morbidity and consume substantially more health care resources than those without [1,2]. Adherence to guidelines and the achievement of treatment targets is associated with better outcomes and a lower rate of resource utilization for patients with chronic diseases [3-5]. For example, tight control of blood pressure in patients with type 2 diabetes leads to a significant increase in time to development of complications, without significantly increasing the total cost of care [3]. Despite guidelines, many people with chronic diseases do not receive recommended care [6-9].

Individuals with chronic diseases may have difficulty achieving care goals due to a combination of patient, provider, and system level barriers [10]. System level barriers include the inherent complexity of the health care system and poor access to primary or specialty care. At the provider level, barriers may include the lack of support systems to implement recommended care. Patient level barriers may include lack of awareness of publicly funded programs including community-based resources, financial constraints, competing priorities (e.g., family and work), and personal circumstances that make following complex care plans particularly challenging. Survey data have demonstrated that barriers for Canadians with a greater number of chronic conditions include being older, less educated, less well off financially, and living in a rural area [2].

Patient navigator programs may help patients with chronic disease achieve their care goals by providing a tailored approach to addressing their specific needs. Patient navigators are trained personnel who aid patients in overcoming barriers to care. The primary role of the navigator is not clinically oriented, and navigators may be nurses, social workers, or lay health workers. They differ from clinical case managers in that they provide no clinical care.

Patient navigator programs were originally established to reduce gaps in cancer care among marginalized populations [11] and are increasingly in use across the United States and Canada [12]. All existing patient navigator programs have the underlying goal of helping patients to overcome modifiable barriers to care to achieve optimal outcomes [13-15]. Depending on the targeted barriers, specific tasks may include one or more of disease education [16,17], health system education [16,18,19], assistance with insurance coverage [20], aid in care coordination [16], and referral to community resources [17], among others.

Reviews by Wells [15] and Paskett [21] of patient navigators in cancer care found that patient navigator programs improved screening adherence rates and suggested that they improved adherence to diagnostic services and improved cancer stage at diagnosis. Other randomized controlled trials indicate that patient navigators improve the rate of enrolment in a community cardiac rehabilitation program [19] and in the completion of steps in the kidney transplant process [22]. However, there is wide variation in the design and implementation of patient navigator programs, and no systematic review to date has summarized the characteristics of successful programs. The purposes of this study are:

1. To assess the effects of patient navigator services, compared with usual care, on the achievement of recommended processes of care and on patient-oriented outcomes in patients with chronic diseases.
2. To explore whether attributes of patient navigator programs explain heterogeneity in effect sizes observed.

## Methods and design

### Types of studies

Included studies will be limited to RCTs, cluster RCTs, and quasi-randomized RCTs.

### Types of participants

#### Inclusion criteria

Studies will be included when the subjects were patients who have or are being screened for one or more chronic conditions including those of acquired infectious etiology and inherited genetic predisposition. The chronic conditions included in Statistics Canada community surveys [23] that are expected to last 6 months or more will be considered, including asthma; arthritis; hypertension; migraine; COPD/emphysema; diabetes; heart disease; cancer; intestinal/stomach ulcers; stroke; urinary incontinence; inflammatory bowel disorder; dementia; mood disorders; anxiety disorders, with the addition of chronic kidney disease, which includes transplant recipients and patients on dialysis; and HIV/AIDS. Participants of all ages will be included and the intervention may be directed to either the patient or their immediate caregivers.

#### Exclusion criteria

Studies with patients without one or more of the chronic diseases listed above will not be included.

### Types of interventions

Studies that examine the effects of a patient navigator intervention will be considered. For the purposes of this review, a patient navigator is a person with or without a health care-related background that engages with patients on an individual basis to determine barriers to accessing care or following recommended guidelines, and provides information relevant to their specific circumstances in order to increase access to components of the health care system and to enhance their chronic disease care. Navigators do not provide clinical advice, but rather focus on helping patients to access, understand, or better utilize available health care resources. Navigators may go by a different name, e.g., *promotoras*, lay health worker, care guide, and so on, and any of these will be included if they meet the above definition. The navigator role must be formalized, as opposed to casual, untrained peer, family, or friend support. Peer support persons or former patients who have been formally trained in a navigator role will be included. The independent initiation of medical treatments or investigations will be considered to be out of scope of patient navigator programs. Professionals carrying out such tasks may be better defined as integrated or clinical case managers [24].

The comparator group will be no intervention, standard or usual care, or interventions of lower intensity, e.g., educational pamphlet. If more than one comparator group is used, we will choose the arm that most closely resembles usual or standard care.

### Outcome measures

Including a diverse group of chronic diseases will lead to predictably heterogeneous outcome measures. We have purposely kept our outcome list broad in order to include all relevant outcomes that may be reported. To facilitate our goal of summarizing the data, we will categorize outcomes into groups characterized by their proximity to mechanism of action of the intervention: patient-level outcomes, intermediate outcomes, and process outcomes. Patient-level measures of interest include mortality, health-related quality of life, and complications of suboptimal disease management such as cardiovascular events. Intermediate measures include disease-specific clinical measures such as glycemic control. Process measures include the expected immediate targets of the intervention such as increased access to appropriate service providers as well as adherence to recommended clinical actions including guideline-concordant use of medications or investigations. Finally, we will assess measures of patient experience and/or patient satisfaction with the navigator intervention.

### Search method

Databases that will be searched are MEDLINE, Embase, the Cochrane Central Register of Controlled Trials (CENTRAL), CINAHL, PsycINFO, and Social Work Abstracts. There will be no date restrictions. The MEDLINE search strategy was peer reviewed *via* PRESS (Appendix).

The search of online databases will include all languages of publication and will be tailored to specific databases by an expert librarian. In addition, the reference lists of included articles and systematic and narrative reviews identified in the electronic search will be reviewed.

Investigators of included published protocols will be contacted for information about other ongoing or unpublished studies.

### Data collection and analysis

Two reviewers will screen titles and abstracts independently, and all studies potentially eligible for inclusion will be retained for full-text review. Two reviewers will then independently screen full-text articles for inclusion or exclusion. Disagreements will be resolved by discussion or with a third reviewer as required.

Data extraction will be done independently by two reviewers using standard data extraction forms; details are presented in Table 1. Original authors will be contacted in writing for requests of any missing data.

**Table 1:** **Description of data extracted from each included study**

**Data**	**Description**
Type of study	RCT, cluster RCT, and quasi-randomized RCT
Study setting	Country, care setting, e.g., community vs. hospital-based
Participant demographics and characteristics	Mean age, percentage female, illness under study
Details of patient navigator services	Professional background of navigators, scope of services, location and frequency of intervention
Control condition and use of co-interventions	
Outcomes assessed	Primary and secondary outcomes as defined by authors
Study duration	Recruitment period, intervention duration, and total study duration
Results	Changes in outcomes
Funding source	Industry vs. other

### Risk of bias assessment

The risk of bias criteria suggested by the Cochrane Effective Practice and Organisation of Care Group (EPOC) will be used to assess the risk of bias in each included study [25]. The tool includes an assessment of bias in nine domains: sequence generation, allocation concealment, baseline outcome measurements between groups, similarity of baseline characteristics, completeness of data, blinding of outcome assessment, protection against contamination, selective outcome reporting, and other. The selective recruitment of cluster participants will be included as an additional domain for cluster RCTs. Two authors will independently assess risk of bias in each domain and report the risk of bias as high, low, or unclear. Disagreements will be resolved by discussion or with a third reviewer as needed.

### Data synthesis

For the primary analysis, all studies with outcomes in the above described categories will be grouped together and results will be summarized by outcome category. A goal of this study is to provide a quantitative assess-ment of the effects of patient navigator services over a predictably heterogeneous group of chronic diseases and outcomes. Therefore, due to anticipated variability in populations and interventions, we will use a stepped approach to data synthesis. If three or more studies report the same outcome within a category, and there are no apparent unit of analysis errors (or intraclass correlation coefficient (ICC) data are available), traditional random effects meta-analysis will be conducted, using standard methods to account for cluster trials as relevant [26,27]. We will report relative risk for dichotomous outcomes and standard mean difference for continuous outcomes. Heterogeneity will be assessed by visual inspection of forest plots, the chi^2^ test, and quantified by the I2 statistic. If significant clinical, methodological, or statistical (I2 value of 50% or more) heterogeneity exists, data will not be pooled.

For outcomes that do not meet these criteria but for which data are dichotomous and where there are three or more studies within a category, we will use a previously described quantitative strategy for reporting median effect sizes [28]. First, for studies with multiple dichotomous outcomes that fall within a category of interest, for example adherence to recommended clinical action, the median effect size - the difference in proportions between the intervention and control - will be calculated for all reported outcomes in that category within that study. The median effect size will represent a single outcome for each study. These data will then be analyzed together and a median effect size and interquartile range across studies will be reported. This approach of using a ‘median of medians’ avoids the use of numerous assumptions to estimate the ICC and avoids skewing by outlying studies. For all other outcomes and circumstances, we will use a narrative approach to data synthesis.

We will pursue exploratory analyses to investigate for differences in outcomes based on the groupings below by stratifying analyses if adequate studies exist in each category. Where median effect sizes were estimated, we will explore potential associations between subgroup characteristics and effect size using a nonparametric Mann-Whitney rank-sum test.

1. Type of disease - cancer, mental health, chronic cardiovascular related diseases (i.e., hypertension, diabetes, cardiovascular disease), other diseases
2. Intensity/type of navigator services - lay navigators, professional navigator, telephone-based, in-person services, embedded within care team, external to care team
3. Patient demographic - adult, pediatric, elderly, >50% with low socioeconomic status, >50% minority patients.

A sensitivity analysis will be done in which we exclude studies at high risk of bias in any of the nine domains listed above. Further, we will perform an additional sensitivity analysis excluding studies focused on disease screening, due to the potential for substantial differences in patient populations and the behavior changes targeted by the intervention.

## Discussion

A comprehensive review of patient navigator programs, including a summary of the elements of programs that are associated with a successful intervention, does not yet exist in the literature. The reviews by Wells *et al*. and Paskett *et al*. found a total of 33 studies on patient navigator programs in cancer that reported on patient outcomes [15,21]. There was wide variation in intervention design, study design, and study quality. In addition, these reviews were restricted to cancer care and did not include a meta-analytic component. The purpose of the present study is to comprehensively and systematically synthesize the evidence of effect of patient navigator interventions on clinical and patient-oriented outcomes in populations across chronic diseases. A thorough and high-quality systematic review of patient navigator programs is needed to 1) translate the current evidence into effective navigator programs, 2) determine key outcomes for future intervention studies, and 3) identify areas for further study. This review will provide important information to those who are contemplating, implementing, or testing a similar intervention.

## Appendix: Literature Search

Ovid MEDLINE® search strategy with no date restrictions.

1. "Continuity of Patient Care"/

2. patient navigation/ or exp patient-centered care/ or case management/

3. ((care or coach* or service* or system*) adj5 (coordinat* or facilitat* or navigat* or transition*)).tw.

4. ((patient* or system* or service*) adj5 (coach* or facilitat* or navigat*)).tw.

5. (guided adj2 care).tw.

6. (case manager* or case management or navigator or navigators).tw.

7. post-discharge support*.tw.

8. 1 or 2 or 3 or 4 or 5 or 6 or 7

9. Chronic Disease/

10. Acquired Immunodeficiency Syndrome/

11. exp Alzheimer Disease/

12. exp Anxiety Disorders/

13. exp Arthritis/

14. exp Asthma/

15. exp Intestinal Diseases/ or exp Irritable Bowel Syndrome/ or exp Colonic Diseases, Functional/or exp Gastrointestinal Diseases/or exp Inflammatory Bowel Diseases/ or exp Crohn Disease/or exp Colitis, Ulcerative/

16. exp Pulmonary Disease, Chronic Obstructive/

17. exp Cerebrovascular Disorders/

18. exp Renal Insufficiency, Chronic/

19. exp Dementia/

20. Depression/

21. exp Depressive Disorder/

22. exp Diabetes Mellitus/

23. exp Emphysema/

24. exp Myocardial Infarction/

25. exp Heart Diseases/

26. exp HIV/ or exp HIV Infections/

27. exp Hypertension/

28. exp Neoplasms/

29. exp Migraine Disorders/

30. exp Mood Disorders/

31. exp Obsessive-Compulsive Disorder/

32. exp Panic Disorder/

33. exp Phobic Disorders/

34. exp Stroke/

35. Ulcer/ or exp peptic ulcer/

36. exp Urinary Incontinence/

37. exp Renal Dialysis/

38. exp Kidney Transplantation/

39. (AIDS or acquired immunodeficiency disorder* or affective disorder* or alzheimer* or agina* or anxiety* or arthritis or asthma or atrial fibrillation or arrhythmia* or bipolar or bladder incontin* or bowel disorder* or brain infarc* or cancer* or carcinoma* or cardiomyopath* or COPD or COAD or cerebral haemorrhage or cerebral hemorrhage or cerebrovascular disease* or chronic airflow obstruction* or chronic condition* or chronic disease* or chronic illness* or chronic kidney disease* or chronic obstructive airway* disease* or chronic obstructive pulmonary disease* or cognitive impair* or combat disorder* or (coronary adj2 disease*) or crohns or dementia or depressi* or diabetes or diabetic* or dialysis or emphysema or gastrointestinal disease* or haemodialysis or heart arrest or heart attack* or heart disease* or heart failure or heart infarction* or hemodialysis or HIV* or human immunodeficiency virus* or human immuno-deficiency virus* or hypertens* or inflammatory bowel disease* or IBD or irritable bowel* or isch?emia* or insulin-depend* or intracranial h?emorrhage* or intra-cranial h?emorrhage* or kidney transplant* or longterm condition* or long-term condition* or longterm disease* or long-term disease* or longterm illness* or long-term illness* or manic disorder* or migraine* or mood disorder* or myocardial infarc* or neoplasia* or neoplastic or neoplasm* or neurosis or neuroses or neurotic or OCD or obsessive compulsive disorder* or obstructive lung disease* or obstructive pulmonary disease* or PTSD or panic attack* or panic disorder* or phobia* or post-trauma or posttrauma or renal dialysis or stroke or tumor* or tumour* or ulcer or ulcers or ulcerative colitis or urinary incontinence or urination disorder* or ventricular fibrillation).tw.

40. 9 or 10 or 11 or 12 or 13 or 14 or 15 or 16 or 17 or 18 or 19 or 20 or 21 or 22 or 23 or 24 or 25 or 26 or 27 or 28 or 29 or 30 or 31 or 32 or 33 or 34 or 35 or 36 or 37 or 38 or 39

41. 8 and 40

42. (randomized controlled trial or controlled clinical trial).pt.

43. drug therapy.sh.

44. (groups or placebo or randomized or randomly or trial).tw.

45. 42 or 43 or 44

46. 41 and 45

47. (animals not humans).sh.

48. 46 not 47

## Abbreviations

CCHS: Canada Community Health Survey
CHF: congestive heart failure
COPD: chronic obstructive pulmonary disease
MI: myocardial infarct
RCT: randomized controlled trial

## References

[1] Broemeling AM, Watson DE, Prebtani F (2008). Population patterns of chronic health conditions, co-morbidity and healthcare use in Canada: implications for policy and practice. Healthc Q.

[2] Health Council of Canada (2011). How do sicker Canadians with chronic disease rate the health care system? Results from the 2011 Commonwealth Fund International Health Policy Survey of Sicker Adults.

[3] UK Prospective Diabetes Study Group (1998). Cost effectiveness analysis of improved blood pressure control in hypertensive patients with type 2 diabetes: UKPDS 40. BMJ.

[4] CDC Diabetes Cost-Effectiveness Group (2002). Cost-effectiveness of intensive glycemic control, intensified hypertension control, and serum cholesterol level reduction for type 2 diabetes. JAMA.

[5] Gray A, Raikou M, McGuire A, Fenn P, Stevens R, Cull C (2000). Cost effectiveness of an intensive blood glucose control policy in patients with type 2 diabetes: economic analysis alongside randomised controlled trial (UKPDS 41): United Kingdom Prospective Diabetes Study Group. BMJ.

[6] Manns BJ, Tonelli M, Zhang J, Campbell DJ, Sargious P, Ayyalasomayajula B (2012). Enrolment in primary care networks: impact on outcomes and processes of care for patients with diabetes. CMAJ.

[7] McAlister FA, Majumdar SR, Eurich DT, Johnson JA (2007). The effect of specialist care within the first year on subsequent outcomes in 24,232 adults with new-onset diabetes mellitus: population-based cohort study. Qual Saf Health Care.

[8] Shah BR, Hux JE, Austin PC (2007). Diabetes is not treated as a coronary artery disease risk equivalent. Diabetes Care.

[9] Toth EL, Majumdar SR, Guirguis LM, Lewanczuk RZ, Lee TK, Johnson JA (2003). Compliance with clinical practice guidelines for type 2 diabetes in rural patients: treatment gaps and opportunities for improvement. Pharmacotherapy.

[10] Ferlie EB, Shortell SM (2001). Improving the quality of health care in the United Kingdom and the United States: a framework for change. Milbank Q.

[11] Freeman HP (2013). The history, principles, and future of patient navigation: commentary. Semin Oncol Nurs.

[12] Walkinshaw E (2011). Patient navigators becoming the norm in Canada. CMAJ.

[13] Parker VA, Lemak CH (2011). Navigating patient navigation: crossing health services research and clinical boundaries. Adv Health Care Manag.

[14] Pedersen A, Hack TF (2010). Pilots of oncology health care: a concept analysis of the patient navigator role. Oncol Nurs Forum.

[15] Wells KJ, Battaglia TA, Dudley DJ, Garcia R, Greene A, Calhoun E (2008). Patient navigation: state of the art or is it science?. Cancer.

[16] Fischer SM, Sauaia A, Kutner JS (2007). Patient navigation: a culturally competent strategy to address disparities in palliative care. J Palliat Med.

[17] Shlay JC, Barber B, Michiewicz T, Maravi M, Drisko J, Estacio R (2011). Reducing cardiovascular disease risk using patient navigators, Denver, Colorado, 2007–2009. Prev Chronic Dis.

[18] Goff SL, Pekow PS, White KO, Lagu T, Mazor KM, Lindenauer PK (2013). IDEAS for a healthy baby–reducing disparities in use of publicly reported quality data: study protocol for a randomized controlled trial. Trials.

[19] Scott LB, Gravely S, Sexton TR, Brzostek S, Brown DL (2013). Examining the effect of a patient navigation intervention on outpatient cardiac rehabilitation awareness and enrollment. J Cardiopulm Rehabil Prev.

[20] Darnell JS (2013). Navigators and assisters: two case management roles for social workers in the Affordable Care Act. Health Soc Work.

[21] Paskett ED, Harrop JP, Wells KJ (2011). Patient navigation: an update on the state of the science. CA Cancer J Clin.

[22] Sullivan C, Leon JB, Sayre SS, Marbury M, Ivers M, Pencak JA (2012). Impact of navigators on completion of steps in the kidney transplant process: a randomized, controlled trial. Clin J Am Soc Nephrol.

[23] Statistics Canada Canadian Community Health Survey 2012. [http://www23.statcan.gc.ca/imdb-bmdi/pub/indexti-eng.htm?MM]. Accessed October, 2013.

[24] Zwarenstein M, Reeves S, Straus SE, Pinfold P, Goldman J. Case management: effects on professional practice and health care outcomes (Protocol). Cochrane Lib. 2011;10.

[25] Effective Practice and Organisation of Care (EPOC). [Suggested risk of bias criteria for EPOC reviews]. EPOC Resources for review authors. Oslo: Norwegian Knowledge Centre for the Health Services; 2015. Available at: http://epoc.cochrane.org/epoc-specific-resources-review-authors.

[26] Rao JN, Scott AJ (1992). A simple method for the analysis of clustered binary data. Biometrics.

[27] Higgins JPT, Green S (2011). Cochrane handbook for systematic reviews of interventions. Version 5.1.0.

[28] Shojania KG, Jennings A, Mayhew A, Ramsay CR, Eccles MP, Grimshaw J (2009). The effects of on-screen, point of care computer reminders on processes and outcomes of care. Cochrane Database Syst Rev.

